# Antiallergic Activity of 6-Deoxy-2-*O*-methyl-6-(*N*-hexadecanoyl)amino-l-ascorbic Acid

**DOI:** 10.3390/molecules26154684

**Published:** 2021-08-03

**Authors:** Kaori Miura, Hiroaki Matsuno, Yuji Iwaoka, Hideyuki Ito, Akihiro Tai

**Affiliations:** 1 LAIMU Corporation, 3-6-12 Shinyokohama, Kohoku-ku, Yokohama, Kanagawa 222-0033, Japan; xiangzhis63@gmail.com; 2 Faculty of Life and Environmental Sciences, Prefectural University of Hiroshima, 5562 Nanatsuka-cho, Shobara, Hiroshima 727-0023, Japan; pine.torchez@gmail.com; 3 Faculty of Health and Welfare Science, Okayama Prefectural University, 111 Kuboki, Soja, Okayama 719-1197, Japan; iwaoka@fhw.oka-pu.ac.jp (Y.I.); hito@fhw.oka-pu.ac.jp (H.I.); 4 Graduate School of Technology, Industrial and Social Sciences, Tokushima University, 2-1 Minamijosanjima-cho, Tokushima 770-8513, Japan

**Keywords:** ascorbic acid derivatives, degranulation, passive cutaneous anaphylaxis, structure-activity relationship

## Abstract

Allergy is an excessive immune response to a specific antigen. Type I allergies, such as hay fever and food allergies, have increased significantly in recent years and have become a worldwide problem. We previously reported that an ascorbic acid derivative having palmitoyl and glucosyl groups, 2-*O*-α-d-glucopyranosyl-6-*O*-hexadecanoyl-l-ascorbic acid (6-sPalm-AA-2G), showed inhibitory effects on degranulation in vitro and on the passive cutaneous anaphylaxis (PCA) reaction in mice. In this study, several palmitoyl derivatives of ascorbic acid were synthesized and a structure–activity relationship study was performed to discover more potent ascorbic acid derivatives with degranulation inhibitory activity. 6-Deoxy-2-*O*-methyl-6-(*N*-hexadecanoyl)amino-l-ascorbic acid (2-Me-6-*N*-Palm-AA), in which a methyl group was introduced into the hydroxyl group at the C-2 position of ascorbic acid and in which the hydroxyl group at the C-6 position was substituted with an *N*-palmitoyl group, exhibited much higher inhibitory activity for degranulation in vitro than did 6-sPalm-AA-2G. 2-Me-6-*N*-Palm-AA strongly inhibit the PCA reaction in mice at lower doses than those of 6-sPalm-AA-2G. These findings suggest that 2-Me-6-*N*-Palm-AA may be a promising therapeutic candidate for allergic diseases.

## 1. Introduction

Type I allergies are an exaggerated response by the immune system centered on mast cells, and atopic dermatitis, atopic bronchial asthma, allergic rhinitis, and anaphylactic shock are typical diseases caused by type I allergies. IgE antibodies bound to high-affinity IgE receptors on the surface of mast cells and basophils in tissues and blood form a crosslinked structure when they interact with antigens, triggering a degranulation reaction [1,2]. This degranulation reaction releases chemical mediators, including histamine and leukotriene, causing various allergic reactions such as smooth muscle contraction and increased vascular permeability. In recent years, the number of allergic patients has been increasing worldwide, and as a countermeasure, there is a need to develop drugs that inhibit degranulation.

**Figure 1:**
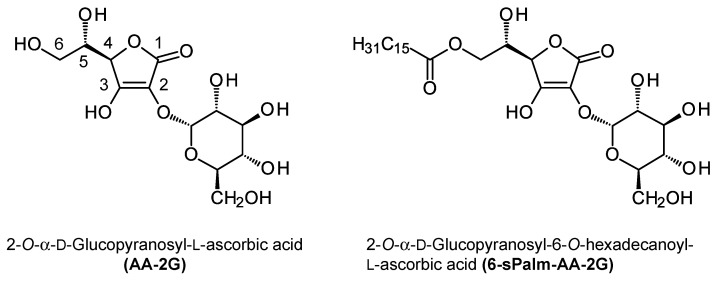
Structures of AA-2G and 6-sPalm-AA-2G.

Ascorbic acid, also known as vitamin C, is one of the water-soluble vitamins that was discovered as an anti-scurvy factor [3]. It has an important function as an antioxidant and is widely used in foods and cosmetics. The enediol structure composed between the hydroxyl groups at the C-2 and C-3 positions of ascorbic acid is highly reactive and causes inactivation and degradation of ascorbic acid itself [4,5]. 2-*O*-α-d-Glucopyranosyl-l-ascorbic acid (AA-2G, Figure 1) is a stable ascorbic acid derivative in which a single molecule of glucose is introduced into the hydroxyl group at the C-2 position of ascorbic acid [6]. AA-2G is an excellent stable provitamin C agent that can be hydrolyzed by α-glucosidase to provide an efficient supply of ascorbic acid [7,8,9,10]. AA-2G is recognized as a food additive and quasi-drug in Japan and is widely used in the fields of food and cosmetics. However, AA-2G has low skin permeability due to its high hydrophilicity [11]. To improve the utilization efficiency of AA-2G as a provitamin C agent, we synthesized a series of stable lipophilic vitamin C derivatives, 6-*O*-acyl-2-*O*-α-d-glucopyranosyl-l-ascorbic acid (6-Acyl-AA-2G) derivatives, having straight-acyl chains or branched-acyl chains of varying lengths [11,12]. 6-Acyl-AA-2G has shown high stability in a neutral solution [11,12], high intestinal absorption [13], and high skin permeability [11,14], and some of its derivatives exhibited more efficient vitamin C activity after being metabolized to ascorbic acid by enzymatic hydrolysis by α-glucosidase and esterase [13,15,16]. These results indicate that 6-Acyl-AA-2G is superior to AA-2G as a provitamin C agent providing ascorbic acid efficiently.

Recently, we found that 6-Acyl-AA-2G exhibits antitumor and antiallergic effects not as a source of vitamin C but as new pharmacological effects. Of the 6-Acyl-AA-2G derivatives, 2-*O*-α-d-glucopyranosyl-6-*O*-(2-propylpentanyl)-l-ascorbic acid (6-bOcta-AA-2G), having a C_8_ branched-acyl chain, suppressed tumor growth in tumor-bearing model mice more strongly than ascorbic acid [17]. The antitumor activity of 6-bOcta-AA-2G is due to the tumor damage caused by 6-*O*-(2-propylpentanyl)-l-ascorbic acid, a metabolic intermediate released by the hydrolysis of 6-bOcta-AA-2G. Additionally, 2-*O*-α-d-glucopyranosyl-6-*O*-hexadecanoyl-l-ascorbic acid (6-sPalm-AA-2G, Figure 1), having a C_16_ straight-acyl chain, showed excellent activity in screening tests for antiallergic activity—namely, evaluation of the degranulation inhibition in RBL-2H3 cells—and inhibited passive cutaneous anaphylaxis (PCA) reactions in mouse skin [18]. It was found that the activity of 6-sPalm-AA-2G was exhibited in the intact form and, furthermore, that the different steric configurations of the glucosyl group at the C-2 position or the hydroxyl group at the C-5 position of the ascorbic acid moiety in 6-sPalm-AA-2G did not affect its activity. However, the chemical structure that is important for the expression of its activity has not yet been clarified.

In this study, in order to clarify the important structures of 6-sPalm-AA-2G for its degranulation inhibitory activity and to create ascorbic acid derivatives with superior antiallergic activity, we synthesized several 6-*O*-palmitoyl-l-ascorbic acid (6-Palm-AA) derivatives and evaluated their structure–activity relationship in degranulation inhibitory effects. A potent derivative was also evaluated for PCA reaction inhibitory activity.

## 2. Results and Discussion

**Figure 2:**
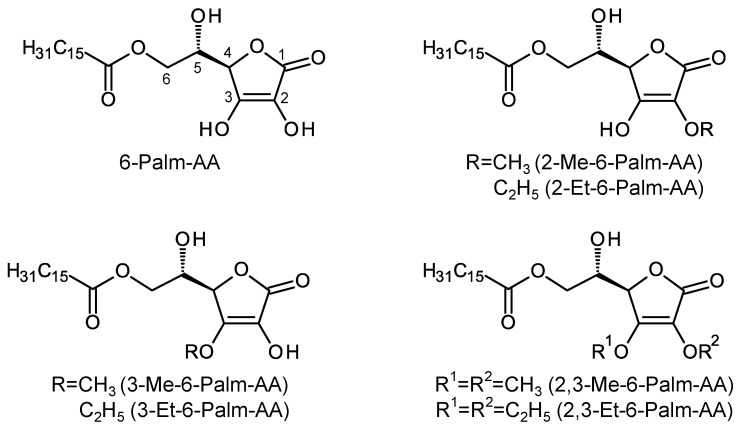
Structures of 6-Palm-AA derivatives.

**Figure 3:**
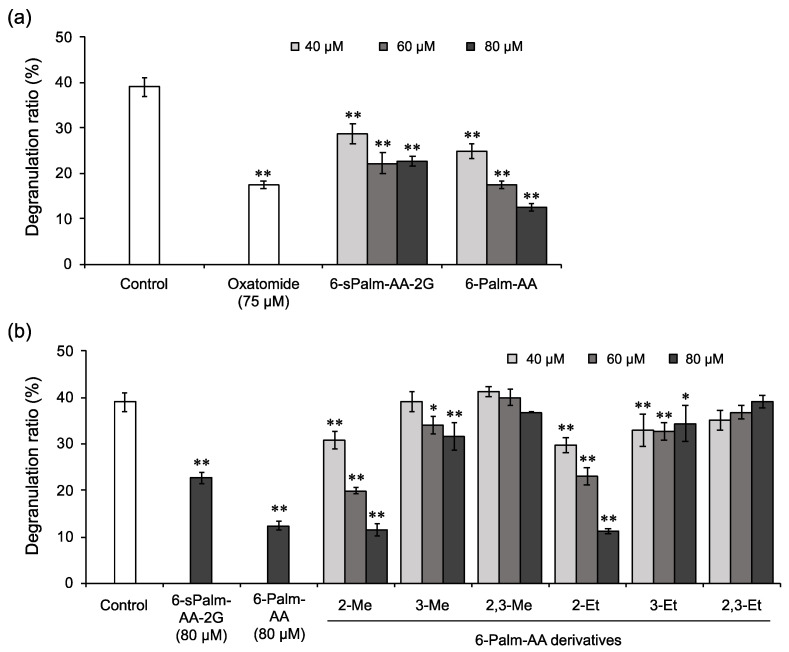
Inhibitory effects of 6-Palm-AA (**a**) and 6-Palm-AA derivatives (**b**) on antigen-induced degranulation. DNP-IgE-sensitized RBL-2H3 cells were incubated with the indicated samples and stimulated with DNP-HSA. All data represent the means ± SD of three independent cultures. * *p* < 0.05 and ** *p* < 0.01 (Dunnett’s test) as compared with the control.

It has been reported that 6-sPalm-AA-2G shows degranulation inhibitory activity and that two isomers with different steric configurations of the glucosyl group at the C-2 position or the hydroxyl group at the C-5 position of the ascorbic acid moiety in 6-sPalm-AA-2G also exhibit the same level of activity [18]. First, to clarify the essential structure for degranulation inhibitory activity of 6-sPalm-AA-2G, the activities of 6-sPalm-AA-2G (Figure 1) and 6-Palm-AA (Figure 2) were compared in RBL-2H3 cells. Oxatomide was used as a positive control. The degranulation inhibitory activity of 6-Palm-AA was stronger than that of 6-sPalm-AA-2G (Figure 3a), while it has already been shown that AA-2G, the depalmitoylated form of 6-sPalm-AA-2G, is inactive [18]. These results indicate that 6-Palm-AA is the essential structure required for degranulation inhibitory activity. Next, the synthesis of various 6-Palm-AA derivatives was devised on the basis of results for the essential structure. Ascorbic acid is very unstable in aqueous solutions [8,19], since the 2,3-enediol moiety of ascorbic acid is highly reactive. 6-Palm-AA is also considered to be unstable. The introduction of at least one substituent into the hydroxyl groups at the C-2 and C-3 positions of ascorbic acid can stabilize the ascorbic acid molecule. Thus, several 6-Palm-AA derivatives were synthesized by introducing a methyl or ethyl group to one or both of the hydroxyl groups at the C-2 and C-3 positions (Figure 2), and their degranulation inhibitory effects on RBL-2H3 cells were evaluated. As shown in Figure 3b, 2-Me-6-Palm-AA and 2-Et-6-Palm-AA substituted with a methyl or ethyl group to the hydroxyl group at the C-2 position showed high degranulation inhibitory activity in a concentration-dependent manner, while 3-Me-6-Palm-AA and 3-Et-6-Palm-AA with substitution at the C-3 position showed reduced degranulation inhibitory activity, and 2,3-Me-6-Palm-AA and 2,3-Et-6-Palm-AA with substitutions at both the C-2 and C-3 positions lost the activity. Since the p*K*a values of ascorbic acid are p*K*a1 of 4.17 and p*K*a2 of 11.57 [20], the hydroxyl group at the C-3 position of ascorbic acid is deprotonated under physiological conditions. These results suggest that the dissociation of the hydroxyl group at the C-3 position of the ascorbic acid moiety is important for degranulation inhibitory activity. The activities of 2-Me-6-Palm-AA and 2-Et-6-Palm-AA at 80 μM were comparable to the activity of 6-Palm-AA and were much higher than the activity of 6-sPalm-AA-2G, an ascorbic acid derivative with a substitution at the same position, while there was no difference in the activities of 2-Me-6-Palm-AA and 2-Et-6-Palm-AA. Judging from the finding that 2-*O*-methyl ascorbic acid is naturally occurring [21] and the desire to find the smallest structure for the activity, 2-Me-6-Palm-AA appeared to be a suitable candidate for further structural development. 

**Figure 4:**
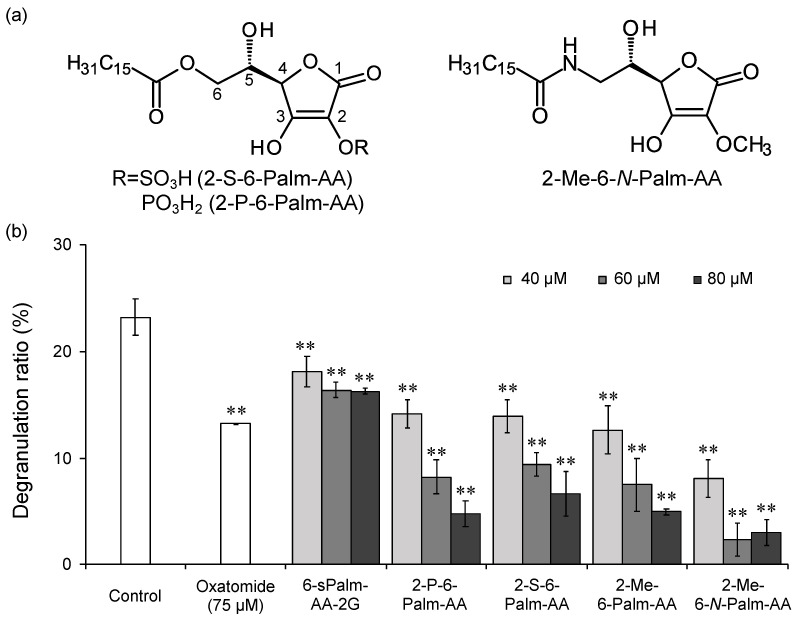
Structures of 6-Palm-AA derivatives with different substituents at the C-2 or C-3 position (**a**) and the inhibitory effects of the 6-Palm-AA derivatives on antigen-induced degranulation (**b**). All data represent the means ± SD of three independent experiments. ** *p* < 0.01 (Dunnett’s test) as compared with the control.

To investigate whether the characteristics and/or the binding mode of a substituent affect the degranulation inhibitory activity, 2-Me-6-Palm-AA analogs were synthesized. There are ascorbic acid 2-phosphate [22] and ascorbic acid 2-sulfate [23] as *O*-substituted ascorbic acid derivatives at the C-2 position. First, 2-P-6-Palm-AA and 2-S-6-Palm-AA (Figure 4a) were synthesized, and their degranulation inhibitory activities were compared with that of 2-Me-6-Palm-AA. 2-Me-6-Palm-AA, 2-P-6-Palm-AA, and 2-S-6-Palm-AA showed almost the same degranulation inhibitory activities (Figure 4b). The substituents at the 2-position of 2-P-6-Palm-AA and 2-S-6-Palm-AA are negatively charged and bonded via an ester bond, while the substituent of 2-Me-6-Palm-AA is not charged and is bonded via an ether bond. The degranulation inhibitory activities of 2-P-6-Palm-AA and 2-S-6-Palm-AA were higher than the activity of 6-sPalm-AA-2G, suggesting that the substituents introduced to the hydroxyl group at the C-2 position must be smaller than the glucose molecule for the exhibition of high activity. These results suggest that relatively small substituents at the 2-position do not affect the activity by the substituent characteristics and binding mode. Next, the degranulation inhibitory activity of 2-Me-6-*N*-Palm-AA (Figure 4a), in which palmitic acid was introduced via an amide bond at the C-6 position, was compared with that of 2-Me-6-Palm-AA. As shown in Figure 4b, 2-Me-6-*N*-Palm-AA exhibited higher activity at all concentrations than 2-Me-6-Palm-AA in which palmitic acid was introduced via an ester bond. The results showed that the binding mode of the palmitoyl group at the 6-position affected the degranulation inhibitory activity. The structure–activity relationship study revealed that the following three factors are important for the degranulation inhibitory activity of 6-Palm-AA derivatives: the existence of a dissociable hydroxyl group at the C-3 position of ascorbic acid, the introduction of a small molecule such as a methyl group into the hydroxyl group at the C-2 position, and the introduction of palmitic acid via an amide bond at the C-6 position. 

**Figure 5:**
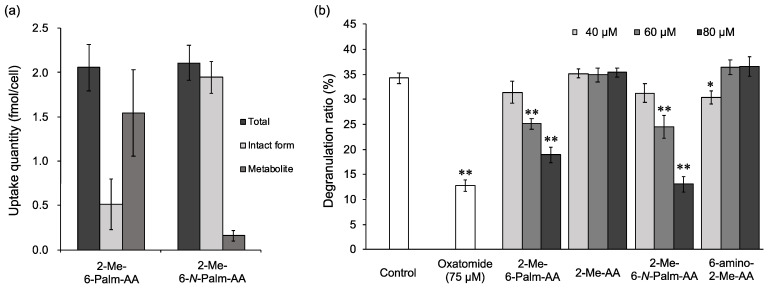
Uptake quantities of 6-Palm-AA derivatives into RBL-2H3 cells (**a**) and the inhibitory effects of 2-Me-6-Palm-AA, 2-Me-6-*N*-Palm-AA, and their metabolites on antigen-induced degranulation (**b**). All data represent the means ± SD of three independent experiments. * *p* < 0.05 and ** *p* < 0.01 (Dunnett’s test) as compared with the control.

To clarify the active body of 2-Me-6-*N*-Palm-AA and 2-Me-6-Palm-AA, the cellular uptake and metabolism of 2-Me-6-*N*-Palm-AA and 2-Me-6-Palm-AA were analyzed, and the activities of their metabolites were compared with those of 2-Me-6-*N*-Palm-AA and 2-Me-6-Palm-AA. 6-Amino-6-deoxy-2-*O*-methyl-l-ascorbic acid (6-amino-2-Me-AA) was detected as a metabolite of 2-Me-6-*N*-Palm-AA, and 2-*O*-methyl-l-ascorbic acid (2-Me-AA) was detected as a metabolite of 2-Me-6-Palm-AA in RBL-2H3 cells. Ascorbic acid was not detected as a metabolite of either of the derivatives. These results suggested that the 6-Palm-AA derivatives were only depalmitoylated in cells. The total cellular uptake amount was expressed as the total amount of the compounds incorporated into the cell as the intact form and its metabolite. 6-Amino-2-Me-AA accounted for 7.7% of the total uptake amount in 2-Me-6-*N*-Palm-AA-treated cells, and 2-Me-AA accounted for 75% of the total uptake amount in 2-Me-6-Palm-AA-treated cells, although the total uptake amounts were the same in both treated cells (Figure 5a). The finding that 2-Me-6-*N*-Palm-AA showed stronger activity than that of 2-Me-6-Palm-AA was thought to be due to the differences in the metabolic susceptibility of the two derivatives to hydrolytic enzymes. Thus, 2-Me-6-*N*-Palm-AA with high degranulation inhibitory activity was less susceptible to be depalmitoylated than 2-Me-6-Palm-AA. 6-Amino-2-Me-AA, the metabolite of 2-Me-6-*N*-Palm-AA, and 2-Me-AA, the metabolite of 2-Me-6-Palm-AA, showed no degranulation inhibitory activity, whereas 2-Me-6-*N*-Palm-AA and 2-Me-6-Palm-AA exhibited significant activity (Figure 5b). These results indicate that the difference between the activities of 2-Me-6-*N*-Palm-AA and 2-Me-6-Palm-AA depends on the amounts of intact derivatives taken up into the cells. Therefore, 2-Me-6-*N*-Palm-AA and 2-Me-6-Palm-AA per se showed degranulation inhibitory activity.

**Figure 6:**
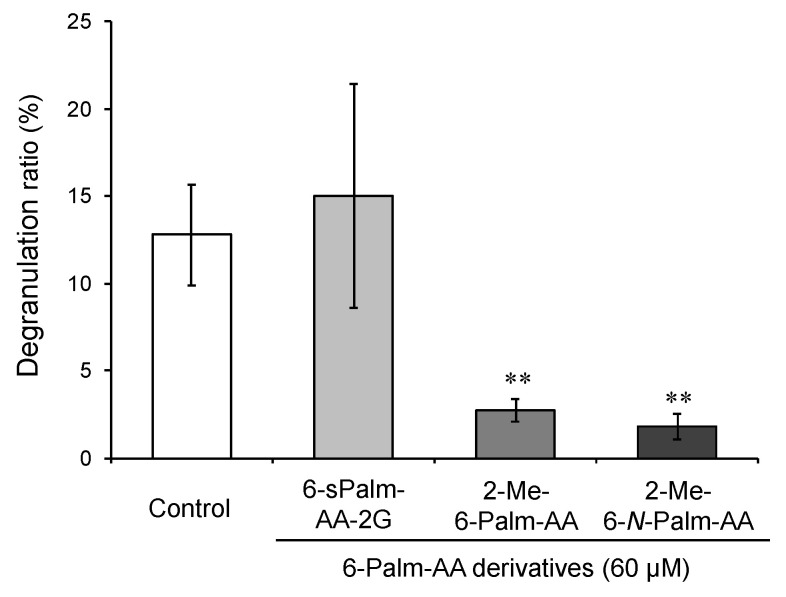
Inhibitory activities of 2-Me-6-Palm-AA and 2-Me-6-*N*-Palm-AA on calcium ionophore A23187-stimulated degranulation in RBL-2H3 cells. All data represent the means ± SD of three independent experiments. ** *p* < 0.01 (Dunnett’s test) as compared with the control.

The degranulation reaction in mast cells and basophils is triggered by the crosslinking of receptor-bound IgE, which results in the phosphorylation of tyrosine, activation of the phosphoinositide 3-kinase, and elevation of intracellular Ca^2+^ levels. Calcium ionophore stimulation, which increases the intracellular Ca^2+^ levels, also causes degranulation [1,2]. In our previous study, we showed that 6-sPalm-AA-2G did not inhibit calcium ionophore A23187-stimulated degranulation but suppressed the phosphorylation of Syk, which plays important roles in early signaling events in antigen-induced degranulation [18]. The inhibitory effects of 2-Me-6-*N*-Palm-AA and 2-Me-6-Palm-AA on degranulation by calcium ionophore A23187 stimulation were examined by comparing them with that of 6-sPalm-AA-2G. 2-Me-6-*N*-Palm-AA and 2-Me-6-Palm-AA significantly inhibited the calcium ionophore stimulation-induced degranulation, but 6-sPalm-AA-2G did not inhibit it (Figure 6). Therefore, the action mechanism in the degranulation inhibitory activity of 2-Me-6-*N*-Palm-AA and 2-Me-6-Palm-AA is considered to be different from that of 6-sPalm-AA-2G.

**Figure 7:**
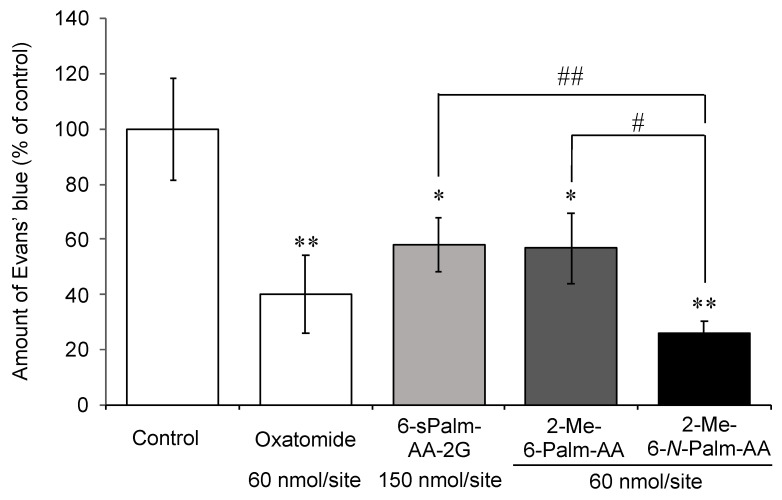
Inhibitory effect of 2-Me-6-*N*-Palm-AA on the antigen-stimulated PCA reaction in mice. Mice were percutaneously administered the indicated samples: control (*n* = 9), oxatomide (*n* = 9), 6-sPalm-AA-2G (*n* = 9), 2-Me-6-Palm-AA (*n* = 7), and 2-Me-6-*N*-Palm-AA (*n* = 8). All data represent the means ± SE. * *p* < 0.05 and ** *p* < 0.01 (Dunnett’s test) as compared with the control. ^#^ *p* < 0.05 and ^##^ *p* < 0.01 (*t*-test).

Finally, we evaluated the inhibitory effect of 2-Me-6-*N*-Palm-AA and, as a comparison, the inhibitory effects of 2-Me-6-Palm-AA and 6-sPalm-AA-2G on the IgE-mediated passive cutaneous anaphylaxis (PCA) reaction in mice. The PCA reaction is a model experiment of the inflammatory reaction of type I allergies in animals, and the PCA reaction in a mouse ear has been used to evaluate various antiallergic effects. Mouse ears were sensitized with IgE antibodies, and antigen and dye were administered intravenously to induce a PCA reaction. The amount of dye leaking from the vessels with increased permeability was measured. Each sample was percutaneously applied to the ears 3.5 h before antigen administration. 2-Me-6-*N*-Palm-AA and 2-Me-6-Palm-AA (60 nmol/site) significantly inhibited the PCA reaction at lower doses than that of 6-sPalm-AA-2G (150 nmol/site) (Figure 7). 2-Me-6-*N*-Palm-AA showed stronger PCA inhibitory activity than that of 2-Me-6-Palm-AA at the same dose. The results may be largely attributed to the fact that 2-Me-6-*N*-Palm-AA is less susceptible to be depalmitoylated than 2-Me-6-Palm-AA, as shown in Figure 5a. In addition, the activity of 2-Me-6-*N*-Palm-AA was comparable or superior to that of oxatomide, an antiallergic drug used for the treatment of allergic diseases such as allergic rhinitis, hives, and atopic dermatitis. The results suggest that 2-Me-6-*N*-Palm-AA will be an effective drug candidate for the treatment of type I allergies.

In the present study, we synthesized several 6-Palm-AA derivatives and identified important structures of 6-Palm-AA derivatives required for degranulation inhibitory activity. In addition, we found that a new 6-Palm-AA derivative, 2-Me-6-*N*-Palm-AA, strongly inhibited the PCA reaction, suggesting that 2-Me-6-*N*-Palm-AA would be useful for the treatment of allergies. Since 2-Me-6-*N*-Palm-AA exhibits degranulation inhibitory activity in its intact form, it is thought that the most efficient antiallergic effect of 2-Me-6-*N*-Palm-AA can be expected by using dosage forms (e.g., eye drops, inhalant, or ointment) that are less susceptible to hydrolytic enzymes. 

## 3. Materials and Methods

### 3.1. General Methods

2-*O*-α-d-Glucopyranosyl-6-*O*-hexadecanoyl-l-ascorbic acid (6-sPalm-AA-2G) was synthesized in our laboratory as described previously [11]. 6-*O*-Palmitoyl-l-ascorbic acid (6-Palm-AA), oxatomide, l-ascorbic acid 2-phosphate trisodium salt, and l-ascorbic acid 2-sulfate disodium salt dihydrate were purchased from FUJIFILM Wako Pure Chemical Corporation, Osaka, Japan. Monoclonal anti-dinitrophenyl antibody (DNP-IgE) and dinitrophenyl-labeled human serum albumin (DNP-HSA) were obtained from Sigma-Aldrich Co., St. Louis, MO, USA. All of the chemicals used were of the highest grade commercially available. NMR spectra were obtained on a Varian NMR System 600-MHz instrument. The values of the chemical shifts were expressed in ppm, and each coupling constant (*J*) was expressed in Hz. The electron spray ionization (ESI) high-resolution mass sptectra were recorded on a Bruker Daltonics (Bremen, Germany) MicrOTOF II instrument using direct sample injection.

### 3.2. Synthesis of 6-O-Hexadecanoyl-2-O-methyl-l-ascorbic Acid (2-Me-6-Palm-AA)

6-Palm-AA (3.11 g, 7.5 mmol) was dissolved in DMF (60 mL), and K_2_CO_3_ (1.04 g, 7.5 mmol) and chloromethyl methyl ether (0.56 mL, 7.5 mmol) were added, and the reaction was carried out at room temperature for 30 min. After the reaction, the solution was separated with EtOAc and 2-M NaCl. The EtOAc layer was washed with 2-M NaCl, dehydrated with Na_2_SO_4_, and concentrated. The resulting 6-*O*-hexadecanoyl-3-*O*-methoxymethyl-l-ascorbic acid (3.16 g, 6.91 mmol) was dissolved in DMSO (60 mL), and K_2_CO_3_ (1.15 g, 8.3 mmol) and iodomethane (0.52 mL, 8.3 mmol) were added, and the reaction was carried out at room temperature for 45 min. After the reaction, the mixture was separated with EtOAc and 2-M NaCl; the EtOAc layer was washed with 2-M NaCl, dehydrated with Na_2_SO_4_, and concentrated. The resulting 6-*O*-hexadecanoyl-2-*O*-methyl-3-*O*-methoxymethyl-l-ascorbic acid (2.98 g, 6.32 mmol) was dissolved in 60% formic acid–30% EtOH–10% H_2_O (100 mL) and reacted at 80 °C for 30 min. After the reaction, the mixture was evaporated and then separated with EtOAc and 2-M NaCl. The EtOAc layer was washed with 2-M NaCl, dehydrated with Na_2_SO_4_, and concentrated. The residue was subjected to Wakogel C-200 (FUJIFILM Wako Pure Chemical Corporation, Osaka, Japan) column chromatography. It was further recrystallized by n-hexane-EtOH to purify 2-Me-6-Palm-AA (1.41 g, 3.29 mmol, 43.9%). ^1^H NMR (600 MHz, CD_3_OD): δ 0.88 (3H, t, *J* = 7.2 Hz), 1.27 (24H, m), 1.61 (2H, qn, *J* = 7.2 Hz), 2.36 (2H, t, *J* = 7.2 Hz), 3.75 (3H, s, H-1′), 4.09 (1H, ddd, *J* = 1.8, 6.0, 7.2 Hz, H-5), 4.16 (1H, dd, *J* = 6.0, 10.8 Hz, H-6a), 4.24 (1H, dd, *J* = 7.2, 10.8 Hz, H-6b), 4.76 (1H, d, *J* = 1.8 Hz, H-4). ^13^C NMR (150 MHz, CD_3_OD): δ 13.0, 22.3, 24.5, 28.8, 28.97, 29.04, 29.2, 29.28, 29.33 (x 3), 29.4 (x 2), 31.7, 33.4, 58.8 (C-1′), 64.2 (C-6), 66.4 (C-5), 75.7 (C-4), 121.9 (C-2), 159.1 (C-3), 171.0, 173.7 (C-1). ^1^H NMR and ^13^C NMR spectra data are shown in the Supplementary Materials (Figures S1 and S2). HRMS (*m*/*z*): [M-H]^−^ calcd for C_23_H_39_O_7_, 427.2701; found, 427.2692. 

### 3.3. Synthesis of 6-O-Hexadecanoyl-3-O-methyl-l-ascorbic Acid (3-Me-6-Palm-AA)

An alkyl group was selectively introduced into the hydroxyl group at the C-3 position of 6-Palm-AA, referring to the method of Nihro et al. [24]. 6-Palm-AA (0.52 g, 1.25 mmol) was dissolved in DMSO (10 mL), and K_2_CO_3_ (0.19 g, 1.38 mmol) and iodomethane (0.085 mL, 1.38 mmol) were added, and the reaction was carried out at room temperature for 45 min. After the reaction, the solution was separated with EtOAc and 2-M NaCl. The EtOAc layer was washed with 2-M NaCl, dehydrated with Na_2_SO_4_, concentrated, and subjected to Wakogel C-200 column chromatography. It was further recrystallized by n-hexane-EtOAc to purify 3-Me-6-Palm-AA (123.4 mg, 0.288 mmol, 23.0%). ^1^H NMR (600 MHz, CD_3_OD): δ 0.89 (3H, t, *J* = 7.2 Hz), 1.29 (24H, m), 1.61 (2H, qn, *J* = 7.2 Hz), 2.35 (2H, t, *J* = 7.2 Hz), 4.01 (1H, ddd, *J* = 1.8, 6.0, 7.2 Hz, H-5), 4.14 (1H, dd, *J* = 6.0, 10.8 Hz, H-6a), 4.17 (3H, s, H-1″), 4.21 (1H, dd, *J* = 7.2, 10.8 Hz, H-6b), 4.69 (1H, d, *J* = 1.8 Hz, H-4). ^13^C NMR (150 MHz, CD_3_OD): δ 13.0, 22.3, 24.5, 28.7, 28.97, 29.05, 29.2, 29.28, 29.33 (x 3), 29.4 (x 2), 31.6, 33.4, 58.3 (C-1″), 64.3 (C-6), 66.5 (C-5), 75.6 (C-4), 119.9 (C-2), 150.6 (C-3), 171.3, 173.7 (C-1). HRMS (*m/z*): [M-H]^−^ calcd for C_23_H_39_O_7_, 427.2701; found, 427.2684.

### 3.4. Synthesis of 2,3-O-Dimethyl-6-O-hexadecanoyl-l-ascorbic Acid (2,3-Me-6-Palm-AA)

6-Palm-AA (0.52 g, 1.25 mmol) was dissolved in DMSO (10 mL), and K_2_CO_3_ (0.19 g, 1.38 mmol) and iodomethane (0.085 mL, 1.38 mmol) were added, and the reaction was carried out at room temperature for 30 min. After the reaction, the solution was separated with EtOAc and 2-M NaCl. The EtOAc layer was washed with 2-M NaCl, dehydrated with Na_2_SO_4_, and concentrated to give crude 3-Me-6-Palm-AA (oil). The 3-Me-6-Palm-AA was dissolved in DMSO (10 mL), and K_2_CO_3_ (0.19 g, 1.38 mmol) and iodomethane (0.085 mL, 1.38 mmol) were added, and the reaction was carried out at room temperature for 30 min. The reaction mixture was separated with EtOAc and 2-M NaCl. The EtOAc layer was washed with 2-M NaCl, dehydrated with Na_2_SO_4_, concentrated, and subjected to Wakogel C-200 column chromatography. Recrystallization with n-hexane-EtOAc gave 2,3-Me-6-Palm-AA (121.4 mg, 0.274 mmol, 21.9%). ^1^H NMR (600 MHz, CD_3_OD): δ 0.88 (3H, t, *J* = 7.2 Hz), 1.29 (24H, m), 1.61 (2H, qn, *J* = 7.2 Hz), 2.35 (2H, t, *J* = 7.2 Hz), 3.78 (3H, s, H-1′), 4.03 (1H, ddd, *J* = 1.8, 6.0, 7.2 Hz, H-5), 4.13 (1H, dd, *J* = 6.0, 10.8 Hz, H-6a), 4.16 (3H, s, H-1″), 4.22 (1H, dd, *J* = 7.2, 10.8 Hz, H-6b), 4.75 (1H, d, *J* = 1.8 Hz, H-4). ^13^C NMR (150 MHz, CD_3_OD): δ 13.0, 22.3, 24.5, 28.7, 28.97, 29.05, 29.2, 29.28, 29.33 (x 3), 29.4 (x 2), 31.6, 33.4, 58.7 (C-1″), 59.7 (C-1′) 64.1 (C-6), 66.4 (C-5), 75.5 (C-4), 122.9 (C-2), 158.1 (C-3), 170.4, 173.6 (C-1). HRMS (*m*/*z*): [M-H]^−^ calcd for C_24_H_41_O_7_, 441.2858; found, 441.2865.

### 3.5. Synthesis of 2-O-Ethyl-6-O-hexadecanoyl-l-ascorbic Acid (2-Et-6-Palm-AA)

6-Palm-AA (1.04 g, 2.5 mmol) was dissolved in DMF (20 mL), and K_2_CO_3_ (0.38 g, 2.75 mmol) and chloromethyl methyl ether (0.21 mL, 2.75 mmol) were added, and the reaction was carried out at room temperature for 30 min. After the reaction, the solution was separated with EtOAc and 2-M NaCl. The EtOAc layer was washed with 2-M NaCl, dehydrated with Na_2_SO_4_, and concentrated to give 6-*O*-hexadecanoyl-3-*O*-methoxymethyl-l-ascorbic acid (1.25 g, 2.73 mmol). 6-*O*-Hexadecanoyl-3-*O*-methoxymethyl-l-ascorbic acid (1.25 g, 2.73 mmol) was dissolved in DMSO (20 mL), and K_2_CO_3_ (0.41 g, 3.00 mmol) and iodoethane (0.24 mL, 3.00 mmol) were added. The reaction was carried out at 50 °C for 30 min. After the reaction, the solution was separated with EtOAc and 2-M NaCl. The EtOAc layer was washed with 2-M NaCl, dehydrated with Na_2_SO_4_, and concentrated. The resulting 2-*O*-ethyl-6-*O*-hexadecanoyl-3-*O*-methoxymethyl-l-ascorbic acid (0.98 g, 2.01 mmol) was dissolved in 60% formic acid–30% EtOH–10% H_2_O (30 mL) and reacted at 80 °C for 30 min. After the reaction, the mixture was evaporated and then separated with EtOAc and 2-M NaCl. The EtOAc layer was washed with 2-M NaCl, dehydrated with Na_2_SO_4_, concentrated, and subjected to Wakogel C-200 column chromatography. 2-Et-6-Palm-AA (407.5 mg, 0.92 mmol, 36.8%) was purified by recrystallization with n-hexane-EtOH. ^1^H NMR (600 MHz, CD_3_OD): δ 0.88 (3H, t, *J* = 7.2 Hz), 1.26 (24H, m), 1.31 (3H, t, *J* = 7.2 Hz, H-2′), 1.61 (2H, qn, *J* = 7.2 Hz), 2.36 (2H, t, *J* = 7.2 Hz), 4.02 (2H, q, *J* = 7.2 Hz, H-1′), 4.09 (1H, ddd, *J* = 1.8, 6.0, 7.2 Hz, H-5), 4.17 (1H, dd, *J* = 6.0, 10.8 Hz, H-6a), 4.24 (1H, dd, *J* = 7.2, 10.8 Hz, H-6b), 4.77 (1H, d, *J* = 1.8 Hz, H-4). ^13^C NMR (150 MHz, CD_3_OD): δ 13.0, 14.0 (C-2′), 22.3, 24.5, 28.8, 28.97, 29.05, 29.2, 29.29, 29.33 (x 3), 29.4 (x 2), 31.6, 33.4, 64.2 (C-6), 66.4 (C-5), 67.3 (C-1′), 75.7 (C-4), 120.4 (C-2), 159.7 (C-3), 171.3, 173.7 (C-1). HRMS (*m*/*z*): [M-H]^−^ calcd for C_24_H_41_O_7_, 441.2858; found, 441.2839.

### 3.6. Synthesis of 3-O-Ethyl-6-O-hexadecanoyl-l-ascorbic Acid (3-Et-6-Palm-AA)

6-Palm-AA (0.52 g, 1.25 mmol) was dissolved in DMSO (10 mL), and K_2_CO_3_ (0.19 g, 1.38 mmol) and iodoethane (0.11 mL, 1.38 mmol) were added, and the reaction was carried out at room temperature for 3 h. After the reaction, the solution was separated with EtOAc and 2-M NaCl. The EtOAc layer was washed with 2-M NaCl, dehydrated with Na_2_SO_4_, concentrated, and subjected to Wakogel C-200 column chromatography. 3-Et-6-Palm-AA (152.9 mg, 0.35 mmol, 28.0%) was purified by recrystallization with n-hexane-EtOAc. ^1^H NMR (600 MHz, CD_3_OD): δ 0.90 (3H, t, *J* = 7.2 Hz), 1.30 (24H, m), 1.36 (3H, t, *J* = 7.2 Hz, H-2″), 1.62 (2H, qn, *J* = 7.2 Hz), 2.36 (2H, t, *J* = 7.2 Hz), 4.02 (1H, ddd, *J* = 1.8, 6.0, 7.2 Hz, H-5), 4.16 (1H, dd, *J* = 6.0, 10.8 Hz, H-6a), 4.23 (1H, dd, *J* = 7.2, 10.8 Hz, H-6b), 4.54 (2H, q, *J* = 7.2 Hz, H-1″), 4.70 (1H, d, *J* = 1.8 Hz, H-4). ^13^C NMR (150 MHz, CD_3_OD): δ 13.0, 14.4 (C-2″), 22.3, 24.6, 28.8, 29.0, 29.1, 29.2, 29.4 (x 6), 31.7, 33.4, 64.3 (C-6), 66.6 (C-5), 67.2 (C-1″), 75.7 (C-4), 119.3 (C-2), 149.8 (C-3), 171.4, 173.7 (C-1). HRMS (*m/z*): [M-H]^−^ calcd for C_24_H_41_O_7_, 441.2858; found, 441.2858.

### 3.7. Synthesis of 2,3-O-Diethyl-6-O-hexadecanoyl-l-ascorbic Acid (2,3-Et-6-Palm-AA)

6-Palm-AA (0.52 g, 1.25 mmol) was dissolved in DMSO (20 mL), and K_2_CO_3_ (0.19 g, 1.38 mmol) and iodoethane (0.11 mL, 1.38 mmol) were added, and the reaction was carried out at 30 °C for 2.5 h. After the reaction, the solution was separated with EtOAc and 2-M NaCl. The EtOAc layer was washed with 2-M NaCl, dehydrated with Na_2_SO_4_, concentrated, and dried to give crude 3-Et-6-Palm-AA (oil). 3-Et-6-Palm-AA was dissolved in DMSO (20 mL), and K_2_CO_3_ (0.19 g, 1.38 mmol) and iodoethane (0.11 mL, 1.38 mmol) were added, and the reaction was carried out at 30 °C for 30 min. After the reaction, the solution was separated with EtOAc and 2-M NaCl. The EtOAc layer was washed with 2-M NaCl, dehydrated with Na_2_SO_4_, concentrated, and subjected to Wakogel C-200 column chromatography. 2,3-Et-6-Palm-AA (59.5 mg, 0.126 mmol, 10.1%) was purified by recrystallization with n-hexane-EtOAc. ^1^H NMR (600 MHz, CD_3_OD): δ 0.88 (3H, t, *J* = 7.2 Hz), 1.28 (24H, m), 1.30 (3H, t, *J* = 7.2 Hz, H-2′), 1.37 (3H, t, *J* = 7.2 Hz, H-2″), 1.61 (2H, qn, *J* = 7.2 Hz), 2.35 (2H, t, *J* = 7.2 Hz), 4.03 (1H, ddd, *J* = 1.8, 6.0, 7.2 Hz, H-5), 4.06 (2H, q, *J* = 7.2 Hz, H-1′), 4.14 (1H, dd, *J* = 6.0, 10.8 Hz, H-6a), 4.22 (1H, dd, *J* = 7.2, 10.8 Hz, H-6b), 4.52 (2H, q, *J* = 7.2 Hz, H-1″), 4.74 (1H, d, *J* = 1.8 Hz, H-4). ^13^C NMR (150 MHz, CD_3_OD): δ 13.0, 14.06 (C-2′), 14.07 (C-2″), 22.3, 24.5, 28.7, 28.97, 29.05, 29.2, 29.28, 29.33 (x 3), 29.4 (x 2), 31.6, 33.4, 64.1 (C-6), 66.5 (C-5), 67.9 (C-1′), 68.1 (C-1″), 75.6 (C-4), 121.2 (C-2), 157.5 (C-3), 170.7, 173.6 (C-1). HRMS (*m/z*): [M-H]^−^ calcd for C_26_H_45_O_7_, 469.3171; found, 469.3184.

### 3.8. Synthesis of 6-O-Hexadecanoyl-2-O-phospho-l-ascorbic Acid (2-P-6-Palm-AA)

2-P-6-Palm-AA was synthesized according to the method of Suzuki et al. [25]. AA-2P·3Na (1.07 g, 3.35 mmol) was reacted with palmitic acid (1.29 g, 5.03 mmol) in H_2_SO_4_ (16.7 mL) for 24 h at room temperature. The reaction mixture was added to ice-water (100 mL) and filtered. The residue was extracted with diethyl ether (140 mL) and washed twice with 2-N HCl (70 mL) containing 30% isopropanol. The diethyl ether layer was dehydrated with Na_2_SO_4_, concentrated, washed twice with n-hexane (30 mL), and filtered under reduced pressure to afford 2-P-6-Palm-AA (0.71 g, 1.44 mmol, 43.2%). ^1^H NMR (600 MHz, CD_3_OD): δ 0.90 (3H, t, *J* = 7.2 Hz), 1.30 (24H, m), 1.62 (2H, qn, *J* = 7.2 Hz), 2.37 (2H, t, *J* = 7.2 Hz), 4.13 (1H, ddd, *J* = 1.8, 6.0, 7.2 Hz, H-5), 4.18 (1H, dd, *J* = 6.0, 10.8 Hz, H-6a), 4.26 (1H, dd, *J* = 7.2, 10.8 Hz, H-6b), 4.84 (1H, d, *J* = 1.8 Hz, H-4). ^13^C NMR (150 MHz, CD_3_OD): δ 13.0, 22.3, 24.5, 28.8, 28.98, 29.05, 29.2, 29.29, 29.33 (x 3), 29.35 (x 2), 31.6, 33.4, 64.1 (C-6), 66.5 (C-5), 75.8 (C-4), 114.0 (C-2), 158.6 (C-3), 169.4, 173.7 (C-1). HRMS (*m/z*): [M-H]^−^ calcd for C_22_H_38_O_10_P, 493.2208; found, 493.2218.

### 3.9. Synthesis of 6-O-Hexadecanoyl-2-O-sulfo-l-ascorbic Acid (2-S-6-Palm-AA)

2-S-6-Palm-AA was synthesized according to the method of Suzuki et al. [25]. AA-2S·2Na·2H_2_O (1.12 g, 3.35 mmol) was reacted with palmitic acid (1.29 g, 5.03 mmol) in H_2_SO_4_ (50 mL) for 24 h at room temperature. The reaction mixture was added to ice-water (100 mL) and filtered. The residue was extracted with diethyl ether (140 mL) and washed twice with 2-N HCl (70 mL) containing 30% isopropanol. The extract was concentrated, washed twice with n-hexane (30 mL), and filtered under reduced pressure to afford 2-S-6-Palm-AA (0.71 g, 1.43 mmol, 43.0%). ^1^H NMR (600 MHz, CD_3_OD): δ 0.90 (3H, t, *J* = 7.2 Hz), 1.31 (24H, m), 1.62 (2H, qn, *J* = 7.2 Hz), 2.37 (2H, t, *J* = 7.2 Hz), 4.08 (1H, ddd, *J* = 1.8, 6.0, 7.2 Hz, H-5), 4.18 (1H, dd, *J* = 6.0, 10.8 Hz, H-6a), 4.25 (1H, dd, *J* = 7.2, 10.8 Hz, H-6b), 4.73 (1H, d, *J* = 1.8 Hz, H-4). ^13^C NMR (150 MHz, CD_3_OD): δ 13.0, 22.3, 24.6, 28.8, 28.98, 29.05, 29.2, 29.29, 29.33 (x 3), 29.4 (x 2), 31.6, 33.4, 64.4 (C-6), 66.6 (C-5), 75.8 (C-4), 118.7 (C-2), 152.6 (C-3), 171.7, 173.7 (C-1). MS (*m/z*): [M+H]^+^ calcd for C_22_H_39_O_10_S, 495.2258; found, 495.2607.

### 3.10. Synthesis of 6-Deoxy-2-O-methyl-6-(N-hexadecanoyl)amino-l-ascorbic Acid (2-Me-6-N-Palm-AA)

2-*O*-Methyl-l-ascorbic acid (2-Me-AA) was synthesized by referring to the method of Kato et al. [26]. Ascorbic acid (100.00 g, 568 mmol) was reacted in acetone (1.0 L) with acetyl chloride (8.5 mL, 119 mmol) as a catalyst for 18 h at room temperature. After the reaction, the crude crystals were collected by filtration under reduced pressure. The resulting 5,6-*O*-isopropylidene-l-ascorbic acid (78.17 g, 362 mmol) was reacted with K_2_CO_3_ (49.97 g, 362 mmol) and chloromethyl methyl ether (27.2 mL, 362 mmol) in 75% THF-DMF (280 mL) at room temperature for 4 h. The reaction was stopped with H_2_O (250 mL) and neutralized with 2-N HCl, and the solution was separated with EtOAc (250 mL). The EtOAc layer was washed with 2-M NaCl, dehydrated with Na_2_SO_4_, and concentrated. The obtained 5,6-*O*-isopropylidene-3-*O*-methoxymethyl-l-ascorbic acid (32.70 g, 127 mmol) was reacted with K_2_CO_3_ (19.1 g, 140 mmol) and iodomethane (8.6 mL, 140 mmol) in 50% THF-DMF (216 mL) at 30 °C for 3.5 h. The reaction was stopped with H_2_O (300 mL) and neutralized with 2-N HCl, and the solution was separated with EtOAc (300 mL). The EtOAc layer was dehydrated with Na_2_SO_4_ and concentrated. The resulting 5,6-*O*-isopropylidene-2-*O*-methyl-3-*O*-methoxymethyl-l-ascorbic acid (22.17 g) was reacted in 25% 2-N HCl-EtOH (110 mL) at 80 °C for 1 h. The reaction solution was concentrated and subjected to DIAION HP20 (Mitsubishi Chemical Corporation, Tokyo, Japan) column chromatography to obtain 2-Me-AA (14.64 g, 76.99 mmol). Amination of the hydroxyl group at the C-6 position of 2-Me-AA was carried out in accordance with the method of Andrews et al. [27]. 2-Me-AA (14.64 g, 76.99 mmol) was reacted with 30% HBr-AcOH (29.7 mL, 154 mmol as HBr) and AcOH (19.1 mL) at 30 °C for 16 h. Then, the reaction solution was concentrated. The residue was reacted with 50% 2-N HCl-EtOH (75 mL) at 60 °C for 3 h. The reaction mixture was concentrated and separated with EtOAc (200 mL) and H_2_O (100 mL). The EtOAc layer was dehydrated with Na_2_SO_4_ and concentrated and then subjected to Wakogel C-200 column chromatography. The obtained 6-bromo-6-deoxy-2-*O*-methyl-l-ascorbic acid (9.31 g, 36.8 mmol) was added to the NaN_3_ (3.59 g, 55.2 mmol)-Na_2_CO_3_ (7.79 g, 73.5 mmol) solution (160 mL) and reacted at room temperature for 15 h. The reaction solution was acidified (ca. pH 4.0) with HCl and subjected to DIAION HP20 column chromatography. The obtained 6-azide-6-deoxy-2-*O*-methyl-l-ascorbic acid (6.00 g, 27.9 mmol) was catalyzed by Pd/C (0.36 g, 0.6% (*w*/*w*)) in H_2_O (100 mL), and hydrogen reduction was carried out for 5 h at room temperature. After the reaction, Pd/C was removed by filtration under reduced pressure and concentrated. The residue was acidified with formic acid (ca. pH 4.0) and subjected to active carbon column chromatography; recrystallization with H_2_O gave 6-amino-6-deoxy-2-*O*-methyl-l-ascorbic acid (6-amino-2-Me-AA) (3.25 g, 17.2 mmol, 3.03%). 6-Amino-2-Me-AA (1.00 g, 5.29 mmol) was reacted with palmitic chloride (3.2 mL, 11.1 mmol) in pyridine (20 mL) at room temperature for 1.5 h. After the reaction, MeOH (20 mL) was added, and the mixture was concentrated. The residue was extracted with MeOH and n-hexane, and the MeOH layer was concentrated. Recrystallization with EtOH gave 2-Me-6-*N*-Palm-AA (1.08 g, 2.53 mmol, 47.8%). ^1^H NMR (600 MHz, CD_3_OD): δ 0.90 (3H, t, *J* = 7.2 Hz), 1.30 (24H, m), 1.61 (2H, qn, *J* = 7.2 Hz), 2.22 (2H, t, *J* = 7.2 Hz), 3.38 (1H, dd, *J* = 6.0, 10.8 Hz, H-6a), 3.43 (1H, dd, *J* = 7.2, 10.8 Hz, H-6b), 3.77 (3H, s, H-1′), 3.98 (1H, ddd, *J* = 1.8, 6.0, 7.2 Hz, H-5), 4.69 (1H, d, *J* = 1.8 Hz, H-4). ^13^C NMR (150 MHz, CD_3_OD): δ 13.0, 22.3, 25.5, 28.9, 29.0, 29.1, 29.2, 29.4 (x 6), 31.7, 35.6, 41.9 (C-6), 58.8 (C-1′), 67.3 (C-5), 76.3 (C-4), 121.8 (C-2), 159.4 (C-3), 171.1, 175.5 (C-1). ^1^H NMR and ^13^C NMR spectra data are shown in the Supplementary Materials (Figures S3 and S4). HRMS (*m*/*z*): [M-H]^−^ calcd for C_23_H_40_NO_6_, 426.2861; found, 426.2866.

### 3.11. Antigen-Mediated Degranulation Assay

Antigen-mediated degranulation assays were performed as described in our previous report [18]. Briefly, RBL-2H3 cells sensitized with anti-DNP-IgE were exposed to each of the test samples for 20 min and then stimulated with DNP-HSA for 1 h. The hexosaminidase activity of the culture supernatant was measured as an indication of degranulation.

### 3.12. Calcium Ionophore-Mediated Degranulation Assay

Calcium ionophore-mediated degranulation assays were performed as described in our previous report [18]. Briefly, RBL-2H3 cells exposed to each of the test samples for 20 min were stimulated with the calcium ionophore A23187 for 1 h. The following experimental procedures were the same as those described above.

### 3.13. Quantification of Intracellular Metabolites 

RBL-2H3 cells were seeded in 60-mm dishes at a density of 3 × 10^6^ cells/6.0 mL/dish and cultured under 5% CO_2_ at 37 °C for 24 h. The cell layer was washed with medium, 5.0 mL of the sample (80 μM) was added, and then, the cells were incubated for 20 min. The supernatant was collected in a centrifuge tube, and the cell layer was washed twice with DPBS (−). The cells were detached using 0.05% trypsin–0.02% EDTA and collected in a centrifuge tube, along with the supernatant, and then centrifuged (500× *g*, 4 °C, 5 min). The supernatant was removed, and ice-cold DPBS (−) (5 mL) was added. The number of cells was counted. The cell layer after centrifugation (10,000× *g* at 4 °C for 10 min) was stored at −80 °C. The cell layer was added to 80% AcCN–50-mM ammonium acetate (200 μL) and sonicated for extraction (0 °C, 5 min). After extraction, the supernatant after centrifugation (10,000× *g*, 4 °C, 10 min) was analyzed by HPLC. Separation for 2-Me-6-Palm-AA and 2-Me-6-*N*-Palm-AA was achieved by the isocratic elution of an Inertsil Ph-3 column (i.d. 4.6 × 100 mm, 3 μm, GL Sciences Inc., Tokyo, Japan) kept at 40 °C with 70% MeOH/H_2_O containing 0.5% formic acid at a flow rate of 0.7 mL/min. The absorbance at 240 nm was monitored. Separation for AA, 2-Me-AA, and 6-Amino-2-Me-AA was achieved by the isocratic elution of an Inertsil Amide column (i.d. 4.6 × 100 mm, 3 μm, GL Sciences Inc., Tokyo, Japan) kept at 40 °C with 80% AcCN–50-mM ammonium acetate at a flow rate of 0.7 mL/min. The absorbance at 260 nm was monitored.

### 3.14. PCA Reaction in Mice

The PCA reaction inhibitory activities were evaluated as described in our previous report [18]. An IgE-induced PCA reaction was carried out as follows. Each mouse (ICR mice, 7 Ws, male) was intradermally injected with 20 μL of anti-DNP-IgE antibody (5 mg/mL) in the ears. After 24 h, each of the test samples was applied to the ears. Each sample was dissolved in ethanol/glycerin/Tween-20/H_2_O = 20/5/0.5/74.5 (*v*/*v*/*v*/*v*). After 3.5 h, the mice received an intravenous injection of saline containing DNP-HSA and Evan’s blue. After 30 min, each ear was removed and dissolved in 1-N KOH solution. The extravasated Evan’s blue dye was extracted with an acetone—0.3-M phosphoric acid (13:5) solution and centrifuged. Absorbance was measured (620 nm), and the percentage of the inhibitory effect on the PCA reaction was calculated.

### 3.15. Data Analysis

In vitro results were expressed as the means and SD, and in vivo results were expressed as the means and SE. Comparison of the two means was performed by the *t*-test. Multiple data comparisons were performed by analysis of variance, followed by Dunnett’s test (* *p* < 0.05 and ** *p* < 0.01).

## 4. Conclusions

In this study, we succeeded in creating a new ascorbic acid derivative, 6-deoxy-2-*O*-methyl-6-(*N*-hexadecanoyl)amino-l-ascorbic acid (2-Me-6-*N*-Palm-AA), that has higher antiallergic activity than that of 6-sPalm-AA-2G, an ascorbic acid derivative that was previously found to have antiallergic activity. The structure–activity relationship study of the 6-Palm-AA derivatives showed that retention of the hydroxyl group at the C-3 position of ascorbic acid is important for expression of the activity and that relatively small molecules such as a methyl group are suitable for substitution of the hydroxyl group at the C-2 position. Furthermore, the introduction of a palmitoyl group via an amid bond was found to result in greater degranulation inhibition and greater PCA reaction inhibition. The results indicate that 2-Me-6-*N*-Palm-AA is an effective drug candidate for the treatment of type I allergies.

## Supplementary Materials

The following are available online, Figure S1: ^1^H NMR spectrum of 2-Me-6-Palm-AA, Figure S2: ^13^C NMR spectrum of 2-Me-6-Palm-AA, Figure S3: ^1^H NMR spectrum of 2-Me-6-*N*-Palm-AA, and Figure S4: ^13^C NMR spectrum of 2-Me-6-*N*-Palm-AA.

## Sample Availability

Samples of the compounds are not available from the authors.
