# Supplementary material for: Antiallergic Activity of 6-Deoxy-2-O-methyl-6-(N-hexadecanoyl)amino-l-ascorbic Acid
Source: Molecules. 2021 Aug 3;26(15):4684. doi: 10.3390/molecules26154684 (PMC8348947; doi:10.3390/molecules26154684)
Supplement: Supplementary file 1 [file molecules-26-04684-s001.zip › molecules-1323775-supplementary.pdf]

Supplementary materials

Anti-allergic activity of 6-deoxy-2-*O*-methyl-6-(*N*-hexadecanoyl)amino-L-ascorbic acid

Kaori Miura [1], Hiroaki Matsuno [2], Yuji Iwaoka [3], Hideyuki Ito [3] and Akihiro Tai [2,4,*]

[1] LAIMU Corporation, 3-6-12 Shinyokohama, Kohoku-ku, Yokohama, Kanagawa 222-0033, Japan; miura@laimu.jp
[2] Faculty of Life and Environmental Sciences, Prefectural University of Hiroshima, 5562 Nanatsuka-cho, Shobara, Hiroshima 727-0023, Japan; q623021jw@ed.pu-hiroshima.ac.jp (H.M.)
[3] Faculty of Health and Welfare Science, Okayama Prefectural University, 111 Kuboki, Soja, Okayama 719-1197, Japan; iwaoka@fhw.oka-pu.ac.jp (Y.I); hito@fhw.oka-pu.ac.jp (H.I.)
[4] Graduate School of Technology, Industrial and Social Sciences, Tokushima University, 2-1 Minami-josanjima, Tokushima 770-8506, Japan
* Correspondence: atai@tokushima-u.ac.jp

Contents:

Figure S1. ^{1}H NMR spectrum of 2-Me-6-Palm-AA

Figure S2. ^{13}C NMR spectrum of 2-Me-6-Palm-AA

Figure S3. ^{1}H NMR spectrum of 2-Me-6-*N*-Palm-AA

Figure S4. ^{13}C NMR spectrum of 2-Me-6-*N*-Palm-AA

^{1}H NMR (CD_3OD, 600 MHz)

2-Me-6-Palm-AA

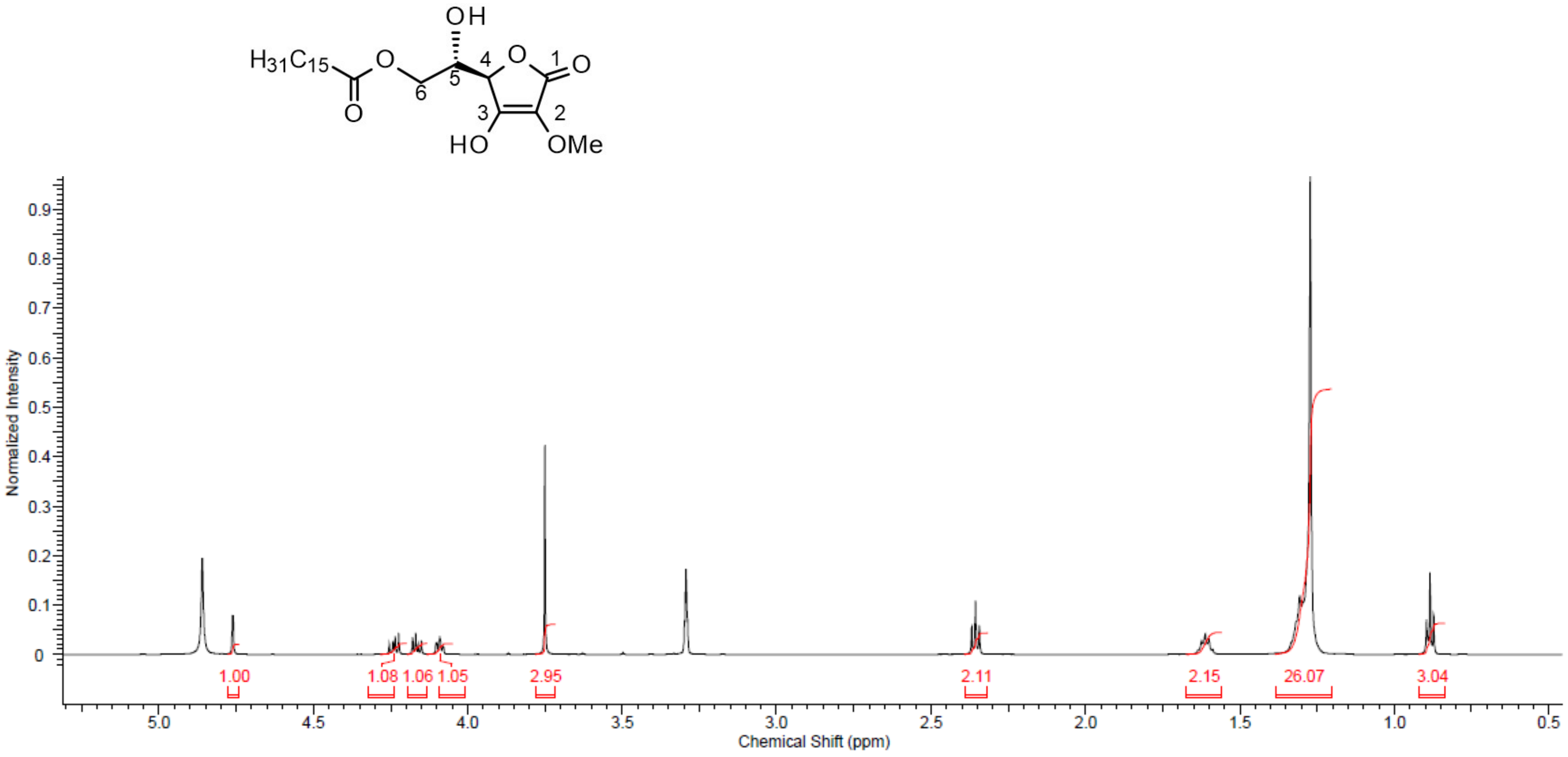

Figure S1. ^{1}H NMR spectrum of 2-Me-6-Palm-AA

^{13}C NMR (CD_3OD, 150 MHz)

2-Me-6-Palm-AA

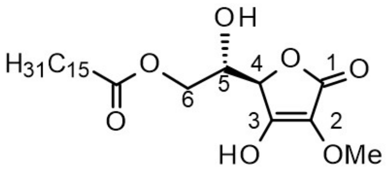

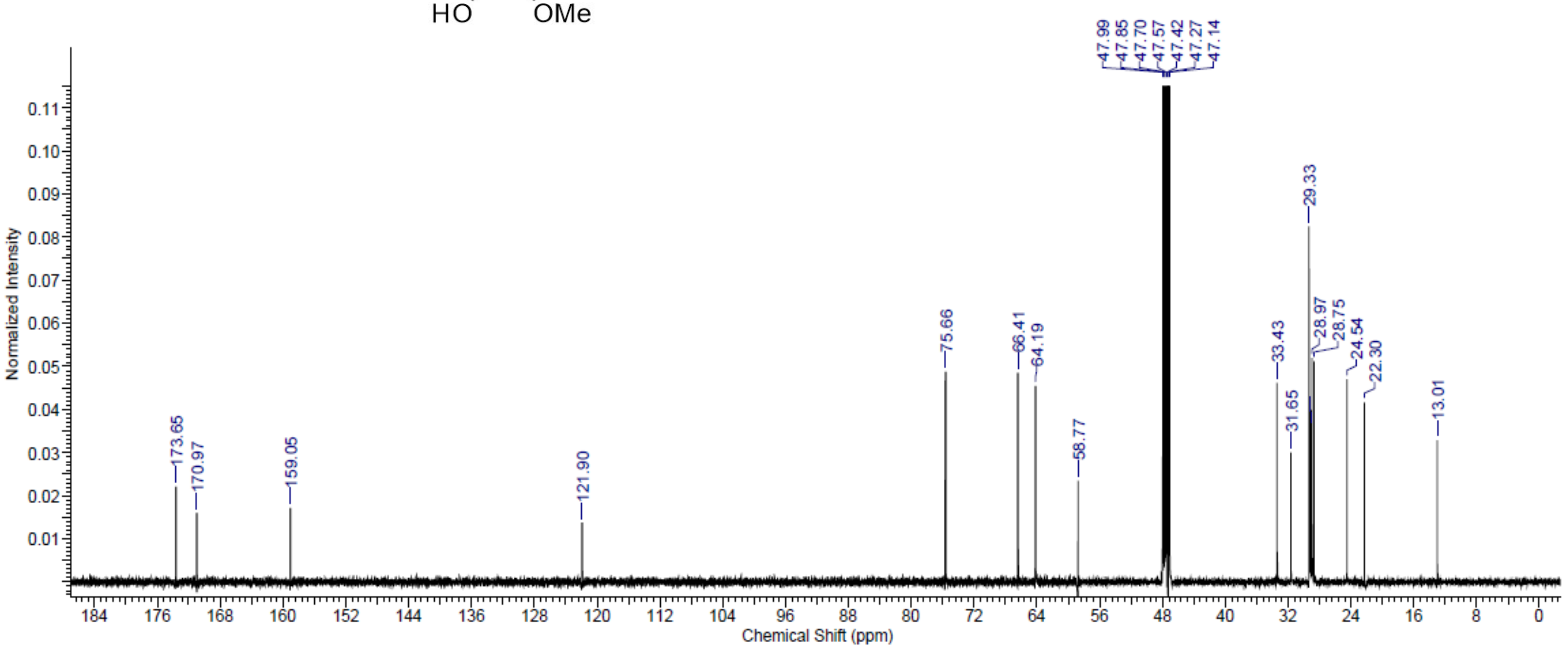

Figure S2. ^{13}C NMR spectrum of 2-Me-6-Palm-AA

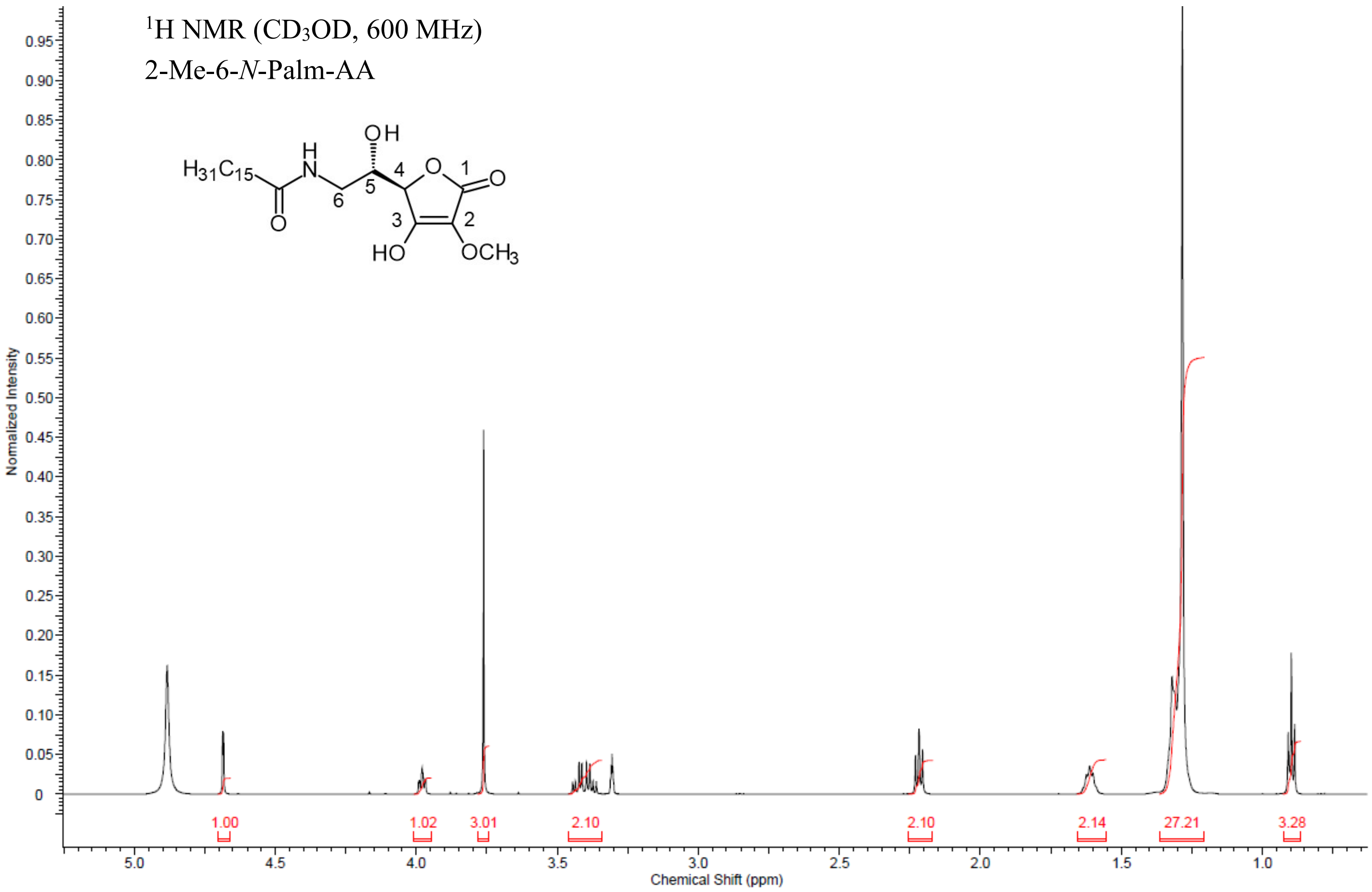

Figure S3. ^{1}H NMR spectrum of 2-Me-6-*N*-Palm-AA

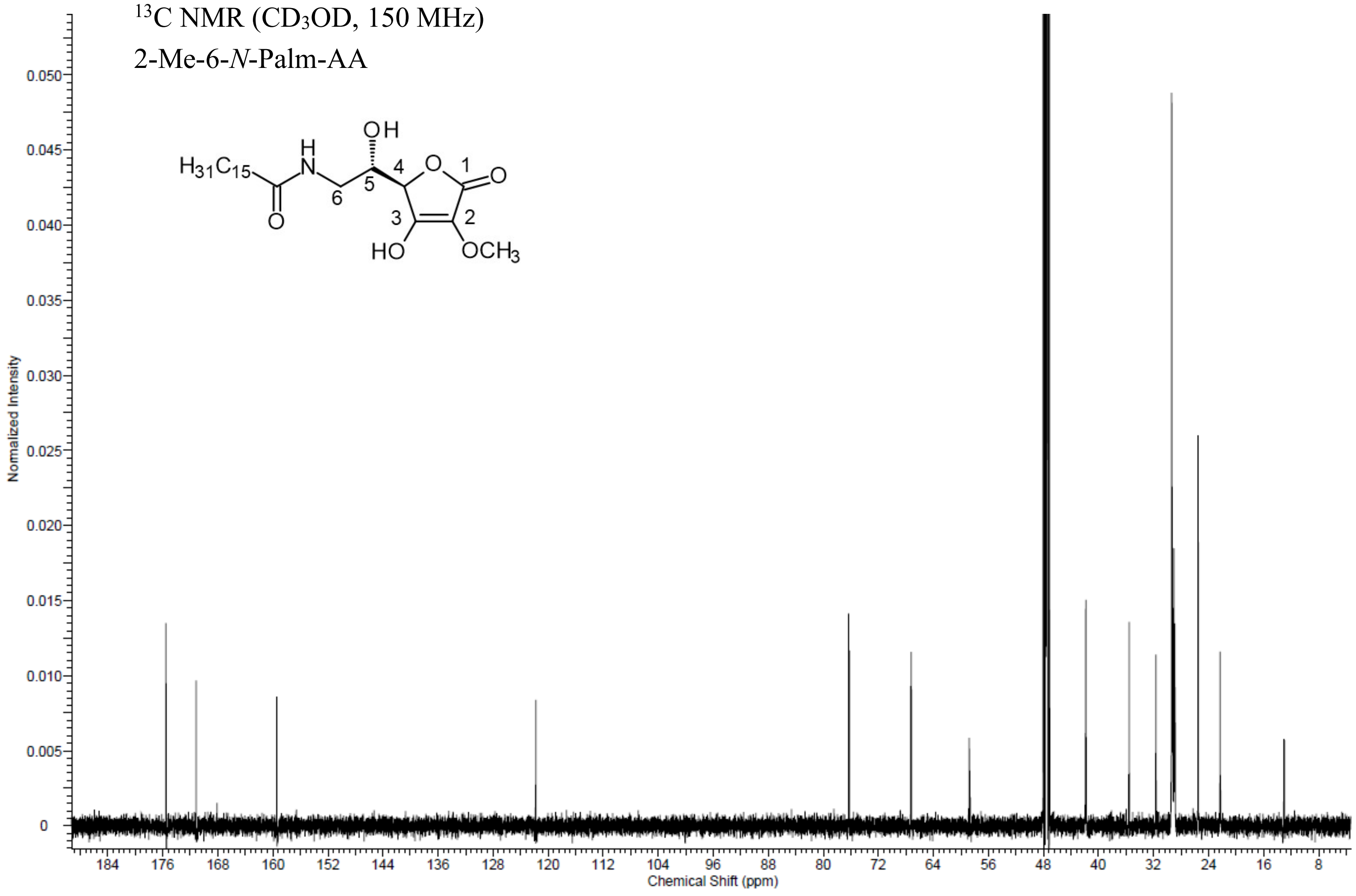

Figure S4. ^{13}C NMR spectrum of 2-Me-6-*N*-Palm-AA
